# Industrial* Trans* Fatty Acid and Serum Cholesterol: The Allowable Dietary Level

**DOI:** 10.1155/2017/9751756

**Published:** 2017-08-30

**Authors:** Hiroyuki Takeuchi, Michihiro Sugano

**Affiliations:** ^1^Department of Food and Nutrition, Toyama College, 444 Mizuguchi, Gankai-ji, Toyama 930-0193, Japan; ^2^Kyushu University, 5-38-23 Najima, Higashi-ku, Fukuoka 813-0043, Japan

## Abstract

*Trans* fatty acid (TFA) from partially hydrogenated oil is regarded as the worst dietary fatty acid per gram due to its role in coronary heart disease. TFA consumption is decreasing worldwide, but some but not all observational studies indicate that TFA intake has little relevance to serum cholesterol levels in populations with low TFA intake (<1%* E* [percentage of total energy intake], <approximately 2 g/day). Few intervention trials examined the effect of TFAs on blood cholesterol at relatively low levels (<2%* E*); no definite evidence is available on the tolerable upper level of the intake. A series of our intervention studies in Japanese suggested that an industrial TFA intake at <1%* E* does not influence the serum cholesterol level. To establish allowable level, we must consider not only the dietary level of TFAs, but also the composition of dietary fats simultaneously consumed, that is, saturated and unsaturated fatty acids. These fatty acids strengthen or counteract the adverse effect of TFAs on serum cholesterol levels. In this review we describe the complex situation of the cardiovascular effects of industrial TFAs. The relationship between dietary industrial TFAs and concentration of plasma cholesterol should be evaluated from the viewpoint of dietary patterns rather than TFAs alone.

## 1. *Trans* Fatty Acid in Foods

*Trans* fatty acid (TFA) is defined as unsaturated fatty acid with at least one nonconjugated double bond in the* trans* configuration. There are several food sources of TFAs. TFA from partially hydrogenated vegetable oil is the major source of dietary industrial TFAs [1], and this type of TFA is regarded as contributing to cardiovascular events. There is a trend toward decreasing consumption of this type of TFA. The second major source of TFA is from ruminant fat, and in some cases ruminant fat is a major contributor of TFAs due to the reduction of the intake of industrial TFAs. The impact of ruminant fat TFAs on human health has not been conclusive, regarding both health benefits [2] and harmful effects [3] depending on the reports. A small amount of industrial TFA is also present in edible oils formed during the deodorization process at high temperature [4]. The physiological effects of TFA contained in edible oils are not well established [5, 6].

Since the TFA in partially hydrogenated oils (PHOs) is composed mainly of a number of positional isomers of the octadecenoic acids, it is important to clarify which TFA isomer(s) is responsible for health hazards. Chatgilialoglu et al. [7] stressed the importance of lipid geometrical isomerism to the biological functions of fatty acids. Ferreri et al. [8] also summarized the significant role of isomerism of fatty acids in membrane functions. In most of the in vitro studies available, elaidic acid (9t-18:1) was examined as a representative TFA in PHO [9]. Elaidic acid is the major component of TFA in PHO, but in many cases it is not always the largest part, usually <30% of the total TFAs. It is plausible that different isomers exert different biological functions, if any. When the principal TFA(s) is revealed, more effective ways to lower the TFA contents of foods can be expected, contributing to human health.

TFA in humans is attributed not only to dietary origin but also to that endogenously formed through the production of free radicals during metabolism [7]. However, it is likely that most of the TFAs in humans are attributable to dietary origin, although the biological activities may differ between these two sources of TFAs. Endogenously formed TFAs were detected in breast cancer tissue specimens [10] and erythrocyte and lymphocyte membranes of children with dermatological diseases [11].

There are several reports concerning the industrial and ruminant TFA content of the foods consumed in various countries [12–17], and Craig-Schmidt and Rong summarized the worldwide consumption of TFAs [18]. In general, the TFA contents of Japanese foods are comparable to those of the corresponding foods in the countries. The amounts of TFA consumed differ among countries, and Japan is probably one of the countries that consumes the least TFA. An example of the industrial and ruminant TFA contents of foods marketed in Japan is shown in Table 1 [19]. The contents of TFA in currently available foods containing partially hydrogenated oils may be somewhat lower than the values shown in this Table, reflecting manufacturers' efforts to reduce TFA contents after the information in the Table was published. However, it should be noted that the industrial TFA content differs widely even in the same foods. Nevertheless, TFA intakes from various foods among Japanese are relatively lower than those in the US and EU, as shown in Table 2 [19]. In the national data, the average intakes of industrial TFA and ruminant TFA in Japanese were estimated to be 0.403 g/day (0.19%* E*) and 0.262 g/day (0.12%* E*), and the 99th percentiles of these values were 1.778 g/day (0.76%* E*) and 1.465 g/day (0.66%* E*) [20].

The measurement of the TFA contents in erythrocytes or plasma should be useful to understand the dietary intake of TFA for estimating the allowable dietary level of TFA as a biomarker [21].

Although the industrial TFA content of vegetable cooking oils without partial hydrogenation is relatively low, vegetable oils are the highest source of dietary TFA among other foods, followed by milk. It is therefore important to determine how much TFAs people in Japan are consuming from each type of food, rather than only the TFA content of the food. The US Food and Drug Administration (FDA) issued a ban in 2015 (applied from June 2018) regarding the use of partially hydrogenated oils [22], and this resulted in a decrease in TFA intake in the US. However, it is impossible to construct healthy diets that are completely free from TFAs, as milk and meat contain TFAs. In light of this situation, it is important to precisely identify the effects of low levels of TFA intake on serum cholesterol levels.

## 2. *Trans* Fatty Acid and Serum Cholesterol

In 1990, Mensink and Katan [23] reported that the consumption of a meal containing TFAs equivalent to 10.9%* E* (percentage of total energy intake) increased the serum LDL-cholesterol and decreased HDL-cholesterol concentrations in healthy subjects. Thereafter, a number of intervention studies have been conducted, and they revealed that a TFA intake above 4%–6%* E* resulted in elevated serum LDL-cholesterol concentrations [24]. Several epidemiological studies provided evidence that the consumption of excess TFAs from industrial sources increases the risk of cardiovascular disease (CVD) [25–27]. Though the influence of excessive industrial TFA intake on both blood lipid levels and the risk of cardiovascular disease has been well established [28], definitive evidence regarding the tolerable upper level of TFA intake does not exist [29].

In the human body, TFAs are metabolized in the same way that* cis* fatty acids are metabolized [30]. TFAs appear to affect serum cholesterol levels through multiple mechanisms including the hepatic production, secretion, and catabolism of circulating lipoproteins [31, 32]. The addition of TFAs increased the secretions of cholesterol [33] and apolipoprotein B-100 [34] by human hepatoma HepG2 cells in vitro. TFA intake increases the plasma activity of cholesterol ester transfer protein (CETP), which is responsible for the transfer of cholesterol esters from high-density lipoprotein (HDL) to low-density lipoprotein (LDL) and very-low-density lipoprotein (VLDL) [35]. These metabolic alterations may at least in part explain the increase of LDL-cholesterol and the decrease of HDL-cholesterol by dietary TFAs.

The World Health Organization (WHO) recommended that TFA intake should be <1%* E* in order to prevent noncommunicable diseases [36]. This level was inferred from the probable safety zone in multivariable regression analyses between TFA intake and the ratio of LDL-/HDL-cholesterol observed in intervention trials [37]. However, the experimental evidence is limited [24]. In fact, the available data concerning the influence of low-level TFA intake (i.e., around 1%* E*) on serum cholesterol levels are insufficient [38].

Since there is a possibility that TFAs and SFAs may be associated with the development of nonalcoholic fatty liver disease (NAFLD) [39], this disease should also be considered as a workable marker for the allowable level of dietary TFAs. There are no definite data available regarding the effect of a low level of TFA on the induction of NAFLD. The current evidence is qualitative. The metabolism of TFA in the hepatocytes may be essentially the same as that of saturated or unsaturated fatty acids, though there are some differences in the oxidation rate. For example, TFA (elaidic acid), compared to oleic acid, was reported to be a better substrate for mitochondrial and peroxisomal oxidation, but a poorer substrate for cellular and very-low-density lipoprotein triacylglycerol synthesis [40].

Although the serum cholesterol level is a good biomarker for CVD risk, attention should also be paid to the biomarkers of systemic inflammation and endothelial dysfunction to confirm the effect of TFA.

## 3. Observation Studies on* Trans* Fatty Acid

Mozaffarian et al. [41] described the relationships between TFA intake and serum lipid levels. In their study, the concentrations of serum LDL- and HDL-cholesterol were measured in 823 generally healthy women living in the US, and the subjects' TFA intake was assessed with the use of a semiquantitative food-frequency questionnaire. The analysis revealed that the subjects' TFA intake was inversely associated with their HDL-cholesterol levels, positively associated with the ratio of LDL- to HDL-cholesterol (Table 3), and not associated with the LDL-cholesterol level. van de Vijver et al. [42] investigated the association between TFA intake and serum lipids in volunteers from eight European countries. They studied 327 male and 299 female apparently healthy volunteers, and TFA intake was assessed using a dietary history. The study results indicated that TFA intake was not associated with the LDL- or HDL-cholesterol levels or the ratio of LDL- to HDL-cholesterol.

The mean TFA intake in the Mozaffarian et al. study was 2.7 g/day (1.3%* E*) and that in the van de Vijver et al. study was 2.2 g/day (0.91%* E*). Thus, the TFA intakes in these studies were almost equal, but there was a clear-cut difference in the association between TFA intake and cholesterol response. These observations suggest the existence of a threshold level causing the different effects of TFAs, but the influence of a difference in the composition of dietary fat should not be excluded.

**Figure 1:**
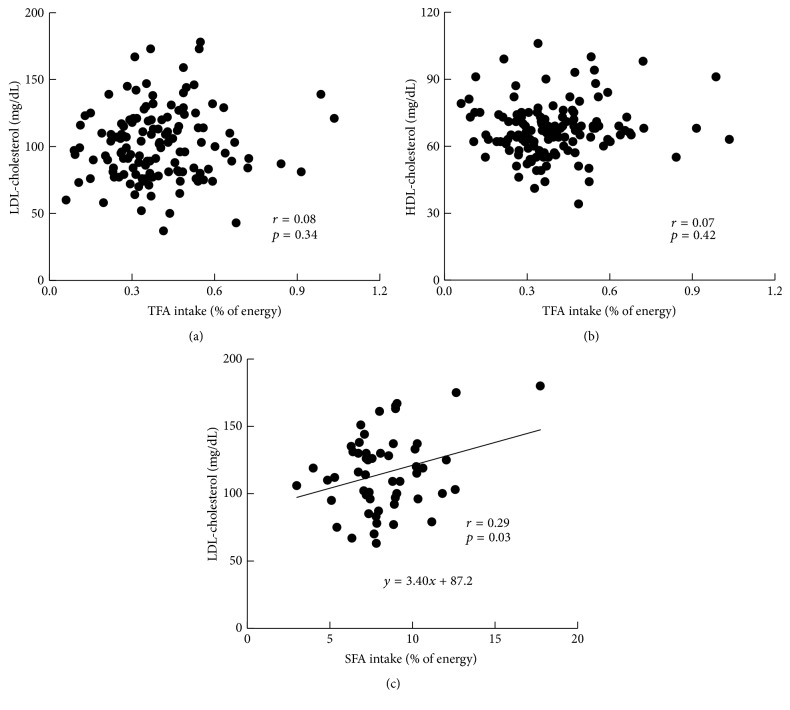
Relationships between the* trans *fatty acid (TFA) intake to LDL-cholesterol (a) and HDL-cholesterol (b), and the relationship between saturated fatty acid (SFA) intake to LDL-cholesterol (c) in 133 young Japanese women [43].

We investigated the relationship between TFA intake and the serum cholesterol levels in 133 young Japanese women [43]. Their TFA intake was assessed with a self-reported written dietary record and a photographic record with a scale card, and the TFA intake was calculated by dietitians using commercially available nutrient calculation software and the data from the Basal Report of Evaluation of TFAs in Food [19]. In this context, the amounts of TFA consumed in the study were more accurate than those in the preceding trials. Our findings revealed a significant correlation between total fat and TFA intakes, whereas TFA intake was not correlated with the total, LDL-, or HDL-cholesterol levels (Figures 1(a) and 1(b)). However, there was a significant correlation between the subjects' saturated fatty acid (SFA) intake and serum LDL-cholesterol levels (Figure 1(c)). The mean intakes of TFA and SFAs were 0.36%* E* and 8.3%* E*, respectively.

These results suggest that the amounts of TFA consumed by young Japanese women may in general not adversely affect their serum cholesterol levels. In light of the relatively low intake of TFA, it appears that more attention should be paid to the intake of SFAs rather than that of TFAs.

Very recently, Yang et al. [44] studied the association between plasma TFA and serum lipid levels before and after the US FDA enacted food-labeling regulations in 2006, and they observed a 54% reduction in plasma TFAs in US adult men and women from 1999-2000 to 2009-2010. Despite the significant reductions, the subjects' plasma TFA concentrations were significantly and consistently associated with serum cholesterol levels. Yang et al. speculated that there does not appear to be a threshold under which the association between the plasma TFA concentration and lipid profiles might become undetectable. The correlation between plasma TFAs and TFA intake is weaker (*r* = 0.30) compared to that between TFAs in erythrocytes and TFA intake (*r* = 0.43) [21], and the content of plasma TFAs may be affected by the serum triacylglycerol concentration. Because subjects with hypertriglyceridemia often have hypercholesterolemia too, considerable attention must be paid to the interpretation of a causal relation between plasma TFA and serum cholesterol levels. The triacylglycerol level in the highest-TFA-quintile group in the Yang et al. study was more than twice that in the lowest-TFA-quintile group (198 mg/dL versus 85.5 mg/dL in 1999-2000, 175 mg/dL versus 74.2 mg/dL in 2009-2010). Because the TFA intake was not described in the Yang et al. study, a direct comparison of our study with their observational study may not be appropriate. In addition, in their study, serum lipid levels were investigated in adult men and women, whereas only female subjects participated in our study.

## 4. Intervention Studies on* Trans* Fatty Acid

A number of intervention studies [31] have demonstrated that industrial TFA at dietary levels above 4%* E*–6%* E* increases blood LDL-cholesterol and reduces HDL-cholesterol. These observations suggest that industrial TFA is more likely to elevate the risk of CHD compared to dietary SFAs, which increase both LDL- and HDL-cholesterol. It was estimated that the intake of TFAs in several European and Asian countries is no more than 2%* E* on average [42, 43, 45], a level that is much lower than the amounts examined in several intervention studies. It is possible that the subgroups in these countries may be consuming higher amounts of industrial TFA, as not all the products are free from TFA even at the present time.

In the US, the intake of industrially produced TFAs decreased substantially after the introduction of the 2003 Nutrition Labeling rule, and the current mean intakes of industrial TFA are estimated to be around 1 g/day, or approx. 0.5%* E* (based on a 2000 kcal daily intake) [46]. However, the number of intervention studies examining the effect on blood cholesterol levels of comparatively low TFA (<2%* E*) is limited [24, 38]. Our summary of six intervention studies assessing the effect of low levels of industrial TFAs is provided as Table 4 [47–52].

In one such study [47], there were no differences in the serum LDL- or HDL-cholesterol levels of moderately hypercholesterolemic subjects who consumed a margarine-containing diet with 3.3%* E* of TFAs and those who consumed a control diet containing 0.55%* E* of TFA over a 5-week period. However, the LDL-cholesterol levels increased after the intake of a diet that included butter (containing 1.3%* E* of TFA) compared to the control diet. Because the content of SFAs in the butter- and margarine-containing diets differed markedly (62% and 25%, resp.), it is difficult to attribute the observed change to TFA alone.

In another study [48], healthy subjects living in the US given a margarine meal containing 1.5%* E* TFAs for 5 weeks had significantly lower LDL-cholesterol levels compared to the values observed after a daily butter meal containing 0.5%* E* from TFAs. Again, there was a detectable difference in the contents of SFAs, and the margarine- and butter-containing diets contained 9%* E* and 16% SFAs. It is plausible that the increase in the LDL-cholesterol due to the butter-containing diet can therefore be attributed more to SFAs than to TFAs. SFAs might have a greater impact than TFAs if the content of TFAs is low in the daily diets on the basis of* E*% of intake.

In order to assess the effect of supplementation with 0.6%* E* or 1%* E* industrial TFAs, we carried out three intervention trials. We conducted a randomized, double-blind crossover trial with two treatment periods of 4 weeks each to assess the effects of 0.6%* E* industrial TFA supplementation on serum cholesterol levels in 12 healthy young Japanese subjects (22.8 ± 3.0 years old) [49]. A 12-week washout period was set between each experimental period. The subjects consumed one cookie containing rapeseed oil (control) or partially hydrogenated rapeseed oil (TFA) every day throughout the treatment periods. The control and TFA cookies contained 0.04 g (0.02%* E*) and 1.13 g (0.6%* E*) of TFAs, respectively. Thus, the difference in dietary fatty acids other than TFAs was negligible in both groups. After the subjects' consumption of the control versus TFA diets, there were no significant between-group differences in the serum concentrations of total, LDL- or HDL-cholesterol. The number of subjects in this study, a total of 12, was too small to draw a conclusion. Larger-scale studies are required.

Under the same protocol [50], we conducted a randomized, double-blind parallel trial to assess the effects of 0.6%* E* industrial TFA supplementation on serum cholesterol levels in healthy adult Japanese women (44.6 ± 4.2 years old). Fifty-one volunteers consumed one cookie containing 0.6%* E* (the TFA diet group) or 0.04%* E* (the control diet group) of TFAs every day for 4 weeks. The volunteers also consumed approx. 0.4%* E* TFAs from their regular meals, and thus the mean TFA intakes of the control and TFA groups during the experimental period corresponded to 0.4%* E* and 1.1%* E*, respectively. Again, there were no significant differences in serum total, LDL- or HDL-cholesterol levels between the control and TFA groups. The results of this trial and our other trial described above [49, 50] indicate that dietary supplementation with 0.6%* E* industrial TFAs (a total TFA intake of approx. 1%* E*) would have little effect on serum cholesterol levels in young and adult healthy subjects.

In a series of trials [51], we addressed the effect of an additional 1%* E* industrial TFA intake on serum cholesterol levels. Sixty-five healthy young Japanese women consumed one cookie a day containing either 1%* E* or 0.04%* E* (control) of TFA for 4 weeks, in addition to their regular meals. The results again showed no significant differences in serum LDL- or HDL-cholesterol levels between the two groups. The results further supported that industrial TFAs at a dietary level of <1%* E* have little effect on serum cholesterol levels in healthy young women.

Indeed, our study protocol may not be appropriate to draw conclusions with respect to the number of participants and may not allow analyses at the subgroup level. However, we observed through three interventions that the plasma cholesterol levels did not change after industrial TFA intake in all participants. The background for this similarity is not apparent.

**Figure 2:**
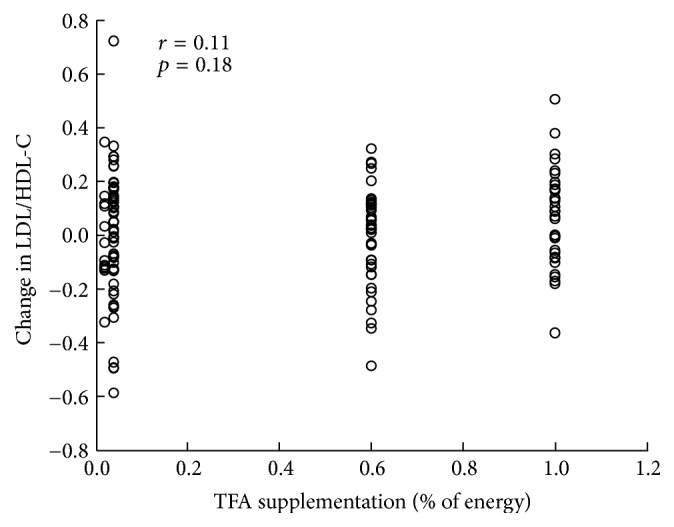
Meta-regression analysis of the change in the ratio of LDL-cholesterol/HDL-cholesterol (LDL/HDL-C) versus the supplementation level of* trans* fatty acids (TFAs) in three intervention trials [49–51].

The results of our meta-regression analysis of changes in the ratio of LDL-/HDL-cholesterol versus the supplementation level of industrial TFAs in our three intervention studies are summarized in Figure 2. We found no significant correlation between the industrial TFA supplementation level and changes in the ratio of LDL-/HDL-cholesterol. The results of these three intervention trials support the soundness of the 2003 WHO recommendation of <1%* E* of TFAs [36].

We have also studied the effects of 1%* E* industrial TFA supplementation on serum cholesterol levels in healthy adults with different obesity-related gene polymorphisms and observed little effect on serum cholesterol levels, regardless of genotype (here, the single nucleotide polymorphism) of fat mass- and obesity-associated gene or beta-3 adrenergic receptor (unpubl. data).

## 5. Linoleic Acid and* Trans* Fatty Acid

Mensink [52] compared the effects of a high-palmitic acid,* trans*-free semiliquid fat with those of a high-oleic acid, low-*trans* semiliquid fat on the serum lipids of healthy subjects. The results indicate that a high-oleic acid, low-*trans *fat has a more favorable impact on the serum lipoprotein profile than a* trans*-free fat high in palmitic acid. Mensink concluded that it is not possible to pinpoint a fat or oil as “good” or “bad” without considering its complete fatty acid composition.

In addition to SFAs, polyunsaturated fatty acids (PUFAs), in particular linoleic acid, favorably influence the blood cholesterol level, a well-known phenomenon commonly accepted in the dietary guidelines regarding the prevention of heart diseases. It has been pointed out that the cholesterol-raising effect of TFAs is attenuated by linoleic acid, as in the case of SFAs [53]. Hu et al. [54] also confirmed in a multivariable analysis of their observation study that the relative risk of cardiovascular disease was lowest when the intake of PUFAs was highest and that of TFAs was lowest. More recently, Hunter [24] proposed that the effect of TFAs on cholesterol levels can be counteracted by the addition of linoleic acid above the 6%* E* level.

Unfortunately, these indications have been almost ignored in the evaluation of the TFA-cholesterol relationship. Since the PUFA/SFA ratio (P/S ratio) of diets in Japanese is considerably high compared to that of people in Western countries, at 2 : 1 versus 1 : 1, it is probable that the expression of the cholesterol-raising effect of TFAs is being attenuated in Japanese. In any case, it is indeed important to consider the complex interaction of dietary fatty acids, not TFA alone but saturated and unsaturated fatty acids too.

## 6. Conclusion

Since the consumption of TFAs is currently decreasing in many countries, it is extremely important to clarify the influence that low dietary levels of TFAs exert on circulating cholesterol levels and cardiovascular diseases. The numbers of investigations of the effects of low-level TFAs on these parameters are insufficient. The results of the few existing studies indicate that TFA at <1%* E* has little adverse effect on the serum cholesterol level. A study conducted in Australia indicated that the relative impact of 0.59%* E* TFA exposure on CHD mortality is limited [55]. On the other hand, another epidemiological study suggested that there does not appear to be a threshold affecting serum lipid levels [44].

Regarding SFAs, although it is generally accepted that an excessive intake of SFAs adversely affects serum cholesterol levels, this conclusion is controversial. The number of the countries that meet the Food and Agriculture Organization (FAO)/WHO recommendation of a mean intake of SFAs of <10%* E* is limited: only 11 of 40 countries reviewed [56], and it is thus necessary to reduce the industrial TFA intake without increasing the SFA intake. A decrease in total fat intake is generally accompanied by a lower intake of not only TFAs but also SFAs. This is the simplest correspondence, but it is also a troublesome approach. Hence, the determination of the tolerable upper level of industrial TFA intake based on reliable evidence is indispensable together with the removal of TFA from foods. In addition, when evaluating the impact of industrial TFAs on our health, it is most important to consider the total dietary pattern, not industrial TFA alone.

## Figures and Tables

**Table 1:** The *trans* fatty acid contents of major foods distributed in Japan.

	Avg.	Max.	Min.	*n*
	g/100 g			
Shortening	13.58	31.21	1.15	10
Margarine and fat spread	7.00	13.49	0.36	34
Creams	3.02	12.47	0.01	10
Butters	1.95	2.21	1.71	13
Biscuits	1.80	7.28	0.04	29
Vegetable oils	1.40	2.78	0.00	22
Animal fats	1.34	2.70	0.64	4
Mayonnaise	1.24	1.65	0.49	9
Cheeses	0.83	1.46	0.48	27
Cakes, buns and pastries	0.71	2.17	0.26	12
Beef	0.52	1.45	0.01	70
Ice creams	0.24	0.60	0.01	14
Japanese buns	0.20	0.34	0.15	4
Breads	0.16	0.27	0.05	5

Reference [19].

**Table 2:** The *trans* fatty acid (TFA) intake from various foods in Japanese.

	Food intake	TFA intake
	g/day	mg/day
Vegetable oils	8.2	114.4
Milk	101.6	92.2
Margarine and fat spread	1.2	84.0
Beef	15.0	78.2
Breads	33.5	54.6
Cakes, buns and pastries	7.4	52.3
Mayonnaise	3.3	40.8
Others of dairy products	8.2	39.5
Biscuits	1.8	32.3
Others of confectioneries	5.3	26.0
Butters	1.1	21.5
Cheeses	2.3	19.0
Japanese buns	6.4	13.1
Fermented milk and lactic acid bacteria beverages	23.1	9.9
Animal fats	0.1	1.4
Others	—	20.8
Total	—	700.0

Reference [19]; TFA intake was calculated from the mean intake and TFA content of each food group. The mean intake of each food group was calculated using the data of National Health and Nutrition Survey carried out for all ages, in a total of 8,762 men and women. To obtain the mean TFA contents of 19 food groups, 386 foods of TFA were determined by gas chromatography.

**Table 3:** Serum LDL- and HDL-cholesterol levels per quintile of *trans* fatty acid (TFA) intake.

	Quintiles of TFA					*p* for trend
	Q1	Q2	Q3	Q4	Q5	
Mozaffarian et al. [41]^1^						
TFA intake, g/d	1.8	2.3	2.7	3.1	3.9	—
LDL-C, mg/dL	118	115	123	118	122	—
HDL-C, mg/dL	70	66	63	63	63	<0.001
LDL:HDL ratio	1.8	1.9	2.1	2.1	2.1	<0.01
van de Vijver et al. [42]^2^						
TFA intake, g/d	0.7	1.4	1.9	2.6	4.4	—
LDL-C, mg/dL	147	154	150	143	143	0.62
HDL-C, mg/dL	58	58	56	58	58	0.27
LDL:HDL ratio	2.72	2.8	2.9	2.7	2.7	0.58

^1^Mean fatty acid intake: saturated; 20 g/day (9.9% *E*), n-6; 10 g/day (5.0% *E*), n-3; 1.2 g/day (0.6% *E*), P/S; 0.56. ^2^Mean fatty acid intake: saturated; 30.4 g/day (12.5% *E*), monounsaturated; 29.3 g/day (12.2% *E*), polyunsaturated; 11.5 g/day (4.7% *E*), P/S; 0.38.

**Table 4:** Summary of six intervention trials that assessed the effect of low-level intakes of *trans* fatty acid (TFA) on serum cholesterol level.

Author (year)	Study design	Baseline demographics	TFA intake, *E*%	SFA intake, *E*%	Weeks	Serum cholesterol level
Lichtenstein et al. (1999) [47]	R, CR, CF	*n* = 36 Healthy, 63 y	Control: 0.55 TFA: 3.30 Butter: 1.25	Control: 7.3 TFA: 8.4 Butter: 16.7	5	Significant LDL-C and Total-C:HDL-C; Control < Butter No significance LDL-C and Total-C:HDL-C; Control versus TFA HDL; Control versus Butter, Control versus TFA

Denke et al. (2000) [48]	CR, CF	*n* = 226 Healthy adult, 41 y Healthy children, 12 y	Butter: 0.9 TFA: 1.5	Butter: 16 TFA: 9	5	Significant LDL-C, Total-C; TFA < Butter No significance HDL-C

Mensink (2008) [52]	CR, CF, DB, R	*n* = 44 Healthy, 41 y	TFA free: 0.2 Low TFA: 0.7	TFA free: 6.2 Low TFA: 2.3	3	Significant LDL-C, HDL-C, Total-C/HDL-C, and Total-C; Low TFA < TFA free

Takeuchi et al. (2011) [49]	CR, DB, R	*n* = 12 Healthy young, 23 y	Control: 0.1 TFA: 0.8	Control: 4.0 TFA: 3.0	4	No significance LDL-C, HDL-C, LDL-C/HDL-C, Total-C

Takeuchi et al. (2013) [51]	DB, P, R	*n* = 65 Healthy young, 18 y	Control: 0.4 TFA: 1.47	Control: 8.7 TFA: 9.8	4	No significance LDL-C, HDL-C, Total-C

Takeuchi et al. (2015) [50]	DB, P, R	*n* = 51 Healthy adult, 45 y	Control: 0.39 TFA: 1.09	Control: 8.0 TFA: 8.3	4	No significance LDL-C, HDL-C, Total-C

CR: crossover; CF: controlled feeding; DB: double-blind; HDL-C: HDL-cholesterol; LDL-C: LDL-cholesterol; P: parallel; R: randomized; SFA: saturated fatty acid; TFA: trans fatty acid; TG: triacylglycerol; Total-C: total cholesterol; y: year.

## References

[1] Mossoba M. M., Moss J., Kramer J. K. G. (2009). *Trans* fat labeling and levels in U.S. foods: Assessment of gas chromatographic and infrared spectroscopic techniques for regulatory compliance. *Journal of AOAC International*.

[2] Dawczynski C., Lorkowski S. (2016). Trans-fatty acids and cardiovascular risk: does origin matter?. *Expert Review of Cardiovascular Therapy*.

[3] Gebauer S. K., Destaillats F., Dionisi F., Krauss R. M., Baer D. J. (2015). Vaccenic acid and trans fatty acid isomers from partially hydrogenated oil both adversely affect LDL cholesterol: A double-blind, randomized controlled trial. *American Journal of Clinical Nutrition*.

[4] De Greyt W., Kellens M., Shahidi F. (2005). Deodorization. *in Bailey's Industrial Oil and Fat Products*.

[5] Wang Q., Imamura F., Lemaitre R. N. (2014). Plasma phospholipid trans-fatty acids levels, cardiovascular diseases, and total mortality: The cardiovascular health study. *Journal of the American Heart Association*.

[6] Wang Q., Imamura F., Ma W. (2015). Circulating and dietary trans fatty acids and incident type 2 diabetes in older adults: The cardiovascular health study. *Diabetes Care*.

[7] Chatgilialoglu C., Ferreri C., Melchiorre M., Sansone A., Torreggiani A. (2014). Lipid geometrical isomerism: from chemistry to biology and diagnostics. *Chemical Reviews*.

[8] Ferreri C., Masi A., Sansone A. (2017). Fatty acids in membranes as homeostatic, metabolic and nutritional biomarkers: recent advancements in analytics and diagnostics. *Diagnostics*.

[9] Itcho K., Yoshii Y., Ohno H. (2017). Association between serum elaidic acid concentration and insulin resistance in two japanese cohorts with different lifestyles. *Journal of Atherosclerosis and Thrombosis*.

[10] Ferreri C., Faraone Mennella M. R., Formisano C., Landi L., Chatgilialoglu C. (2002). Arachidonate geometrical isomers generated by thiyl radicals: The relationship with trans lipids detected in biological samples. *Free Radical Biology and Medicine*.

[11] Ferreri C., Angelini F., Chatgilialoglu C. (2005). Trans fatty acids and atopic eczema/dermatitis syndrome: The relationship with a free radical cis-trans isomerization of membrane lipids. *Lipids*.

[12] Pérez-Farinós N., Dal Re Saavedra M. Á., Villar Villalba C., Robledo de Dios T. (2016). Trans-fatty acid content of food products in Spain in 2015. *Gaceta Sanitaria*.

[13] Otite F. O., Jacobson M. F., Dahmubed A., Mozaffarian D. (2013). Trends in trans fatty acids reformulations of US supermarket and brand-name foods from 2007 through 2011. *Preventing chronic disease*.

[14] Arcand J., Scourboutakos M. J., Au J. T. C., L'Abbe M. R. (2014). Trans Fatty acids in the Canadian food supply: An updated analysis. *American Journal of Clinical Nutrition*.

[15] McCarthy J., Barr D., Sinclair A. (2008). Determination of trans fatty acid levels by FTIR in processed foods in Australia. *Asia Pacific Journal of Clinical Nutrition*.

[16] Becker W., Eriksson A., Haglund M., Wretling S. (2015). Contents of total fat, fatty acids, starch, sugars and dietary fibre in Swedish market basket diets. *British Journal of Nutrition*.

[17] Kris-Etherton P. M., Lefevre M., Mensink R. P., Petersen B., Fleming J., Flickinger B. D. (2012). Trans fatty acid intakes and food sources in the U.S. population: NHANES 1999-2002. *Lipids*.

[18] Craig-Schmidt M. C., Rong Y., Destaillats F., Sébédio J-L., Dionisi F., Chardigny J-M. (2009). Chapter 13 Evolution of worldwide consumption of trans fatty acids. *Trans Fatty Acids in Human Nutrition*.

[19] Food Safety Commission in Cabinet Office (2007). Basal report of evaluation of *trans* fatty acids in food. *General Research of Food Safety*.

[20] Food Safety Commission in Cabinet Office (2012). Estimation of *trans* fatty acid intake. *Trans Fatty Acids in Food*.

[21] Sun Q., Ma J., Campos H., Hankinson S. E., Hu F. B. (2007). Comparison between plasma and erythrocyte fatty acid content as biomarkers of fatty acid intake in US women. *The American Journal of Clinical Nutrition*.

[22] Smith E. D., Turhollow, Zimmerman G. P., Eaton L. M., Bast C. B. (2015). Food and Drug Administration. *Final determination regarding partially hydrogenated oils*.

[23] Mensink R. P., Katan M. B. (1990). Effect of dietary trans fatty acids on high-density and low-density lipoprotein cholesterol levels in healthy subjects. *New England Journal of Medicine*.

[24] Hunter J. E. (2006). Dietary trans fatty acids: Review of recent human studies and food industry responses. *Lipids*.

[25] Willett W. C., Stampfer M. J., Manson J. E. (1993). Intake of trans fatty acids and risk of coronary heart disease among women. *The Lancet*.

[26] Ascherio A., Hennekens C. H., Buring J. E., Master C., Stampfer M. J., Willett W. C. (1994). Trans-fatty acids intake and risk of myocardial infarction. *Circulation*.

[27] Oomen C. M., Ocké M. C., Feskens E. J. M., Van Erp-Baart M.-A. J., Kok F. J., Kromhout D. (2001). Association between trans fatty acid intake and 10-year risk of coronary heart disease in the Zutphen Elderly Study: A prospective population-based study. *Lancet*.

[28] Uauy R., Aro A., Clarke R. (2009). Who scientific update on trans fatty acids: Summary and conclusions. *European Journal of Clinical Nutrition*.

[29] Trumbo P. R., Shimakawa T. (2011). Tolerable upper intake levels for trans fat, saturated fat, and cholesterol. *Nutrition Reviews*.

[30] Emken E. (2013). Human studies using isotope labeled fatty acids: Answered and unanswered questions. *Journal of Oleo Science*.

[31] Mozaffarian D., Katan M. B., Ascherio A., Stampfer M. J., Willett W. C. (2006). Trans fatty acids and cardiovascular disease. *New England Journal of Medicine*.

[32] Kwon Y. (2016). Effect of trans–fatty acids on lipid metabolism: Mechanisms for their adverse health effects. *Food Reviews International*.

[33] Dashti N., Feng Q., Freeman M. R., Gandhi M., Franklin F. A. (2002). Trans polyunsaturated fatty acids have more adverse effects than saturated fatty acids on the concentration and composition of lipoproteins secreted by human hepatoma HepG2 cells. *Journal of Nutrition*.

[34] Mitmesser S. H., Carr T. P. (2005). Trans fatty acids alter the lipid composition and size of apoB-100-containing lipoproteins secreted by HepG2 cells. *Journal of Nutritional Biochemistry*.

[35] van Tol A., Zock P. L., van Gent T., Scheek L. M., Katan M. B. (1995). Dietary trans fatty acids increase serum cholesterylester transfer protein activity in man. *Atherosclerosis*.

[36] World Health Organization (2003). Overall goals. *Diet, Nutrition and the Prevention of Chronic Diseases: Report of a joint WHO/FAO expert consultation*.

[37] Ascherio A., Katan M. B., Zock P. L., Stampfer M. J., Willett W. C. (1999). Trans fatty acids and coronary heart disease. *New England Journal of Medicine*.

[38] Liska D. J., Cook C. M., Wang D. D., Gaine P. C., Baer D. J. (2016). Trans fatty acids and cholesterol levels: An evidence map of the available science. *Food and Chemical Toxicology*.

[39] Ferolla S. M., Silva L. C., Ferrari M. D. L. A. (2015). Dietary approach in the treatment of nonalcoholic fatty liver disease. *World Journal of Hepatology*.

[40] Guzmán M., Klein W., Gómez Del Pulgar T., Geelen M. J. H. (1999). Metabolism of trans fatty acids by hepatocytes. *Lipids*.

[41] Mozaffarian D., Pischon T., Hankinson S. E. (2004). Dietary intake of trans fatty acids and systemic inflammation in women. *American Journal of Clinical Nutrition*.

[42] van de Vijver L. P. L., Kardinaal A. F. M., Couet C. (2000). Association between trans fatty acid intake and cardiovascular risk factors in Europe: The TRANSFAIR study. *European Journal of Clinical Nutrition*.

[43] Takeuchi H., Ito E., Tomioka T., Tabuchi E., Fuhshuku K.-I., Asano Y. (2012). Trans fatty acid intake and serum cholesterol levels in young japanese women. *Bioscience, Biotechnology and Biochemistry*.

[44] Yang Q., Zhang Z., Loustalot F. (2017). Plasma trans-fatty acid concentrations continue to be associated with serum lipid and lipoprotein concentrations among US adults after reduction in trans-fatty acid intake. *Journal of Nutrition*.

[45] Liu A. D., Li J. W., Liu Z. P. (2015). *Trans* Fatty Acid Levels in Foods and Intakes among Population Aged 3 Years and above in Beijing and Guangzhou Cities, China. *Biomedical and environmental sciences*.

[46] Doell D., Folmer D., Lee H., Honigfort M., Carberry S. (2012). Updated estimate of *trans* fat intake by the US population. *Food additives and contaminants. Part A, Chemistry, analysis, control, exposure and risk assessment*.

[47] Lichtenstein A. H., Ausman L. M., Jalbert S. M., Schaefer E. J. (1999). Effects of different forms of dietary hydrogenated fats on serum lipoprotein cholesterol levels. *New England Journal of Medicine*.

[48] Denke M. A., Adams-Huet B., Nguyen A. T. (2000). Individual cholesterol variation in response to a margarine- or butter-based diet: A study in families. *Journal of the American Medical Association*.

[49] Takeuchi H., Yamaki M., Hirose K., Hienae C., Tabuchi E., Sugano M. (2011). Effect of a 0.6% energy trans fatty acid intake on serum cholesterol concentrations in healthy young Japanese subjects. *Bioscience, Biotechnology and Biochemistry*.

[50] Takeuchi H., Nishimura Y., Ohmori A., Tabuchi E. (2015). Little effect of supplementation with 0.6% energy Trans fatty acids on serum cholesterol levels in adult Japanese women. *Journal of Nutritional Science and Vitaminology*.

[51] Takeuchi H., Kutsuwada T., Shirokawa Y., Harada S., Sugano M. (2013). Supplementation with 1% energy trans fatty acids had little effect on serum cholesterol levels in healthy young Japanese women. *Bioscience, Biotechnology and Biochemistry*.

[52] Mensink R. P. (2008). Effects of products made from a high-palmitic acid, trans-free semiliquid fat or a high-oleic acid, low-trans semiliquid fat on the serum lipoprotein profile and on C-reactive protein concentrations in humans. *European Journal of Clinical Nutrition*.

[53] Kritchevsky D. (1997). Trans fatty acids and cadiovascular risk. *Prostaglandins Leukotrienes and Essential Fatty Acids*.

[54] Hu F. B., Stampfer M. J., Manson J. E. (1997). Dietary fat intake and the risk of coronary heart disease in women. *The New England Journal of Medicine*.

[55] Wu J., Zheng M., Catterall E. (2017). Contribution of Trans-Fatty Acid Intake to Coronary Heart Disease Burden in Australia: A Modelling Study. *Nutrients*.

[56] Harika R. K., Eilander A., Alssema M., Osendarp S. J. M., Zock P. L. (2013). Intake of fatty acids in general populations worldwide does not meet dietary recommendations to prevent coronary heart disease: A systematic review of data from 40 countries. *Annals of Nutrition and Metabolism*.

